# High Risk of Incident-Persistent Human Papillomavirus Infection With 9-Valent Vaccine Types in Young Men With an HPV Infection

**DOI:** 10.1093/ofid/ofag045

**Published:** 2026-02-02

**Authors:** Cara J Broshkevitch, Joseph E Tota, Joel M Palefsky, Georges J Nahhas, Stephen E Goldstone, Brady Dubin, Alfred Saah, Alain Luxembourg, Christine Velicer, Anna R Giuliano

**Affiliations:** Department of Epidemiology, Biostatistics and Research Decision Sciences, Merck Research Laboratories, Merck & Co., Inc., Rahway, New Jersey, USA; Department of Epidemiology, Biostatistics and Research Decision Sciences, Merck Research Laboratories, Merck & Co., Inc., Rahway, New Jersey, USA; Department of Medicine, University of California, San Francisco, California, USA; Department of Epidemiology, Biostatistics and Research Decision Sciences, Merck Research Laboratories, Merck & Co., Inc., Rahway, New Jersey, USA; Department of Surgery, Icahn School of Medicine at Mount Sinai, New York, New York, USA; Merck Research Laboratories, Merck & Co., Inc., Rahway, New Jersey, USA; Global Medical and Scientific Affairs, Merck & Co., Inc., Rahway, New Jersey, USA; Merck Research Laboratories, Merck & Co., Inc., Rahway, New Jersey, USA; Merck Research Laboratories, Merck & Co., Inc., Rahway, New Jersey, USA; Department of Cancer Epidemiology, Center for Immunization and Infection Research in Cancer, Moffitt Cancer Center and Research Institute, Tampa, Florida, USA

**Keywords:** HPV vaccines, human papillomavirus, incident infections, males, persistent infections

## Abstract

**Background:**

Studies evaluating the association between prevalent human papillomavirus (HPV) infection and the risk of future incident-persistent infection in a broad global sample of men are needed.

**Methods:**

Heterosexual men (HM) and men who have sex with men (MSM) aged 16–27 years in the placebo arm of a multinational 4-valent HPV vaccine trial (NCT00090285) were assessed. Incident-persistent infection was defined as a new 9-valent (9v) HPV (HPV6/11/16/18/31/33/45/52/58) type at follow-up that was not present at the same anatomic site at baseline and that remained detectable at that same site for 2 or more consecutive visits approximately 6 months apart. Association between baseline prevalent 9vHPV infection and incident-persistent infection at anogenital sites was estimated using incidence rate differences (IRDs) and incidence rate ratios (IRRs).

**Results:**

Included were 1459 HM and 260 MSM. Incidence rates (per 100 person-years) of incident-persistent infection among HM with or without baseline prevalent 9vHPV infection were 10.18 and 5.94, respectively, and among MSM were 11.74 and 9.78, respectively. Baseline prevalent 9vHPV infection status was associated with incident-persistent 9vHPV infection among HM (IRD, 4.24 [95% CI, 1.24–7.23]; adjusted IRR, 1.52 [1.11–2.09]) but not MSM (IRD, 1.96 [−4.01 to 7.93]; adjusted IRR, 1.02 [0.59–1.79]).

**Conclusions:**

HM with baseline prevalent 9vHPV infection were more likely to develop an incident-persistent anogenital infection than those without baseline prevalent infection. Risk of incident-persistent infection among MSM was high regardless of baseline prevalent 9vHPV infection status. These findings highlight the importance of vaccinating men against HPV infection.

Anogenital human papillomavirus (HPV) infection is common in men and is the cause of most cases of anal and penile cancer [1, 2]. The epidemiology and natural history of male HPV infection was evaluated in the large, prospective, multinational HPV infection in men (HIM) study, in which men (aged 18–70 years) residing in Brazil, Mexico, and the United States were enrolled [1]. Analysis of the HIM study showed high incidence rates of new genital HPV infections in a combined population of men who have sex with men (MSM) and men who have sex with women (46.1 per 100 person-years) [3] and of new anal HPV infections among MSM (31.1 per 100 person-years) [4].

Currently available HPV vaccines are exclusively prophylactic and therefore provide maximum benefit when administered prior to initiation of sexual activity and elevated risk of exposure to HPV. Therefore, in most countries, HPV-naive children and adolescents (aged 9–14 years) are the recommended target population for routine HPV vaccination [5, 6]. Evidence suggests, however, that unvaccinated men with a prevalent HPV infection and who have not been vaccinated as adolescents [7, 8] may also be a group for whom to prioritize vaccination. Studies to evaluate the association between prevalent HPV infection and risk of future incident different-type HPV infections in a broad global sample of men are necessary.

We assessed the association between baseline prevalent 9-valent (9v) HPV infection status and incident-persistent anogenital 9vHPV infection among heterosexual men (HM) and MSM. This is a post hoc analysis of global data from the placebo arm of a global, randomized, 4-valent (4v) HPV vaccine efficacy trial [9].

## METHODS

### Data Source

Data were analyzed from sexually active young men (HM and MSM) enrolled in the placebo arm of a global, randomized controlled trial of the 4vHPV vaccine (V501-020 trial; NCT00090285) [9]. The V501-020 trial was sponsored by Merck Sharp & Dohme LLC, a subsidiary of Merck & Co., Inc., Rahway, NJ, USA, and was conducted in accordance with principles of Good Clinical Practice and was approved by the appropriate institutional review boards and regulatory agencies. All participants (or their legally authorized representatives) provided written informed consent at the start of the study. Details of the V501-020 trial have been previously reported [9].

Briefly, key inclusion criteria were HM (aged 16–23 years) who had 1–5 lifetime exclusively female sex partners or MSM (aged 16–26 years) with 1–5 lifetime sex partners who reported engaging in either insertive or receptive anal intercourse, or oral sex, with another male partner within the past year [9]. In addition, HM and MSM must have had no previous diagnosis of HIV infection, no evidence of anogenital lesions suggesting non-HPV sexually transmitted disease, and no clinical evidence or history of anogenital warts or dysplasia [9]. HM and MSM diagnosed with HIV before the first day of the study were excluded; during follow-up, HM from South Africa and all MSM underwent HIV serological testing annually, and HM from other countries were only tested if clinically indicated. MSM residing in South Africa were not enrolled [9].

### Human Papillomavirus DNA Sampling and Assessment

On day 1 (baseline), at month 7, and at 6-month intervals throughout the follow-up period, penile, scrotal, and perineal/perianal swabbed specimens were obtained from all study participants (HM and MSM), and intra-anal specimens were obtained from MSM. We used results from HPV testing of all specimens by multiplex polymerase chain reaction (PCR)-based assays [10, 11] to identify DNA for the 9 HPV types targeted by the 9vHPV vaccine (6/11/16/18/31/33/45/51/52/58).

### Analysis Population and Assessments

The analysis population comprised all participants randomly assigned to the placebo arm of the V501-020 trial who had a day 1 PCR result (negative or positive) for all 9vHPV types from swabbed samples at each anogenital site and had at least 2 follow-up visits with a PCR test result for any 9vHPV type. A *prevalent infection* was defined by a positive PCR test result at any sampled site for any 9vHPV type at the day 1 study visit. *No prevalent infection* was defined by a negative PCR test result for all 9vHPV types at all sampled sites at the day 1 study visit. An *incident-persistent infection* was defined by detection of a 9vHPV type at any sampled site that was not present at the same site at baseline and that remained detectable at that same site for 2 or more consecutive visits approximately 6 months apart (within a 1-month window for each visit). Throughout the analysis, penile and scrotal 9vHPV test results were grouped together as a single penile/scrotal anatomical site. Similarly, perineal and perianal 9vHPV test results were grouped together as a single perineal/perianal anatomical site.

### Statistical Analysis

Incidence rates of incident-persistent 9vHPV infection were calculated per 100 person-years among participants who had at least 2 follow-up visits with a PCR test result for any 9vHPV type. Results were stratified by prevalent 9vHPV infection status at baseline. Incidence rate differences (IRDs) and incidence rate ratios (IRRs) were calculated to estimate the association between prevalent 9vHPV infection at baseline and incidence of incident-persistent 9vHPV infection. IRRs were adjusted for age, geographic region, and number of lifetime female (HM) or male (MSM) sex partners in a multivariable Poisson regression model with a log link. Separate models were developed for HM and MSM. Results were further stratified by type group of incident-persistent infection: HPV6, HPV11, HPV16, HPV16/18, any 4vHPV, or any 9vHPV type.

The cumulative incidence of incident-persistent 9vHPV infection was estimated over 36 months using the Kaplan–Meier method among participants who had at least 2 follow-up visits with a PCR test result for any 9vHPV type. Results were stratified by prevalent 9vHPV infection status at baseline and type group of incident-persistent infection: HPV6, HPV11, HPV16, HPV16/18, any 4vHPV, or any 9vHPV type.

All analyses were stratified by sexual orientation (HM or MSM) and included participants from all regions. In the main analysis, only penile/scrotal and perineal/perianal anogenital site infections were considered for both HM and MSM (ie, intra-anal site infections among MSM were excluded). A separate analysis that included intra-anal site infections among MSM was conducted.

## RESULTS

### Demographic and Behavioral Characteristics of Heterosexual Men and Men Who Have Sex With Men

There were 1459 HM and 260 MSM included in this analysis. Prevalent 9vHPV infection at baseline was more common among MSM (29.6% [77/260]) than HM (13.2% [192/1459]). Irrespective of sexual orientation, demographic variables were generally well balanced for participants with or without prevalent 9vHPV infection, although distributions of the geographic region at enrollment differed (Table 1). Overall, 74.5% of HM and 70.1% of MSM who had prevalent 9vHPV infection at baseline were aged 15–19 years at the first instance of intercourse. More HM with prevalent 9vHPV infection at baseline had 4 or 5 lifetime female sex partners (47.4%) than HM without prevalent 9vHPV infection at baseline (28.8%). Substantial proportions of MSM with or without prevalent infection had 4 or 5 lifetime male sex partners (55.8% and 39.3%, respectively), 3–6 lifetime partners with insertive anal intercourse (55.8% and 36.6%), and 3–6 lifetime partners with receptive anal intercourse (48.1% and 37.2%).

**Table 1.** Baseline Demographic and Behavioral Characteristics, Stratified by Participant Sexual Orientation and Baseline Prevalent 9vHPV Infection Status Among HM and MSM Excluding Intra-Anal Infections^a^

	HM n = 1459		MSM n = 260	
	No 9vHPV Infection at Baseline, No. (%) n = 1267	Prevalent 9vHPV Infection at Baseline, No. (%) n = 192	No 9vHPV Infection at Baseline, No. (%) n = 183	Prevalent 9vHPV Infection at Baseline, No. (%) n = 77
Age, y				
16–20	722 (57.0)	94 (49.0)	54 (29.5)	26 (33.8)
21–27	545 (43.0)	98 (51.0)	129 (70.5)	51 (66.2)
Geographic region				
North America	263 (20.8)	27 (14.1)	88 (48.1)	16 (20.8)
Latin America	558 (44.0)	94 (49.0)	38 (20.8)	26 (33.8)
Europe	149 (11.8)	24 (12.5)	33 (18.0)	21 (27.3)
Asia-Pacific	117 (9.2)	5 (2.6)	24 (13.1)	14 (18.2)
Africa	180 (14.2)	42 (21.9)	NA	NA
Tobacco use on day 1				
Never used	726 (57.3)	107 (55.7)	101 (55.2)	31 (40.3)
Ex-users	78 (6.2)	13 (6.8)	22 (12.0)	6 (7.8)
Current user	463 (36.5)	72 (37.5)	60 (32.8)	40 (51.9)
Age at first occurrence of intercourse, y				
< 15	146 (11.5)	38 (19.8)	22 (12.0)	12 (15.6)
15–19	101 (79.9)	143 (74.5)	114 (62.3)	54 (70.1)
≥ 20	107 (8.4)	11 (5.7)	38 (20.8)	11 (14.3)
Frequency of condom use in the past 6 m				
Always	449 (35.4)	53 (27.6)	67 (36.6)	36 (46.8)
More than half the time	241 (19.0)	51 (26.6)	38 (20.8)	21 (27.3)
Less than half the time	177 (14.0)	31 (16.1)	15 (8.2)	7 (9.1)
Never	370 (29.2)	53 (27.6)	50 (27.3)	13 (16.9)
Number of new female partners in past 6 m				
0	764 (60.3)	108 (56.3)	41 (22.4)	21 (27.3)
1	410 (32.4)	63 (32.8)	2 (1.1)	1 (1.3)
≥2	90 (7.1)	21 (10.9)	1 (0.5)	NA
Number of lifetime female sex partners				
0–3	900 (71.0)	101 (52.6)	43 (23.5)	22 (28.6)
4 or 5	365 (28.8)	91 (47.4)	1 (0.5)	NA
Number of new male partners in past 6 m				
0	NA	NA	59 (32.2)	28 (36.4)
1	NA	NA	71 (38.8)	29 (37.7)
≥2	NA	NA	41 (22.4)	20 (26.0)
Number of lifetime male sex partners				
0–3	NA	NA	99 (54.1)	34 (44.2)
4 or 5	NA	NA	72 (39.3)	43 (55.8)
Number of lifetime partners with which participant had insertive anal intercourse				
0	NA	NA	17 (9.3)	13 (16.9)
1	NA	NA	49 (26.8)	11 (14.3)
2	NA	NA	37 (20.2)	10 (13.0)
3–6	NA	NA	67 (36.6)	43 (55.8)
Number of lifetime partners with which participant had receptive anal intercourse				
0	NA	NA	20 (10.9)	4 (5.2)
1	NA	NA	38 (20.8)	15 (19.5)
2	NA	NA	44 (24.0)	21 (27.3)
3–6	NA	NA	68 (37.2)	37 (48.1)

Abbreviations: 9vHPV, 9-valent human papillomavirus; HM, heterosexual men; HPV, human papillomavirus; MSM, men who have sex with men; NA not available.

^a^Only penile/scrotal and perineal/perianal infections were considered.

### Association Between Prevalent Human Papillomavirus Infection at Baseline and Incidence of Human Papillomavirus Infection at Follow-Up

Among HM, the incidence (per 100 person-years) of incident-persistent 9vHPV infection was 10.18% among those with prevalent 9vHPV infection at baseline compared with 5.94% among those with no prevalent 9vHPV infection at baseline (Table 2). A similar trend was observed for incident-persistent 4vHPV infection (6.81% and 3.62%, respectively). The incidence of incident-persistent 9vHPV infection among MSM (excluding intra-anal site infections) was 11.74% among those with prevalent 9vHPV infection at baseline compared with 9.78% among those with no prevalent 9vHPV infection at baseline. Incidences of incident-persistent 4vHPV infections were 5.57% and 8.18%, respectively. Prevalent 9vHPV infection status at baseline was associated with an increased risk of incident-persistent 9vHPV infection among HM (IRD [per 100 person-years], 4.24 [95% CI, 1.24–7.23]; adjusted IRR, 1.52 [95% CI, 1.11–2.09]) but not MSM (IRD, 1.96 [95% CI, −4.01 to 7.93]; adjusted IRR, 1.02 [95% CI, .59–1.79]). In addition, prevalent 9vHPV infection status at baseline was associated with an increased risk of incident-persistent 4vHPV infection among HM (IRD, 3.18 [95% CI, .77–5.59]; adjusted IRR, 1.75 [95% CI, 1.18–2.60]) but not MSM (IRD, −2.61 [95% CI, −7.02 to 1.81]; adjusted IRR, 0.56 [95% CI, .27–1.17]). Among HM and MSM, associations between prevalent infection status at baseline and the risk of incident-persistent HPV16 or HPV16/18 infections were not statistically significant (Table 2).

**Table 2.** Incidence (per 100 Person-Years) of Incident-Persistent External Genital HPV Infection by Prevalent 9vHPV Infection Status at Baseline Among HM (Aged 16–24 Years)

Prevalent 9vHPV Infection Status at Baseline	HPV Type Group of Incident-Persistent Infection	Participants at Risk, No.	Incident-Persistent Infections, No.	Incidence Per 100 Person-Years, %	IRD (95% CI)	IRR (95% CI)	Adjusted IRR^a^ (95% CI)
**HM (n = 1459)**							
Prevalent 9vHPV infection	9vHPV	192	48	10.18	4.24 (1.24–7.23)	1.72 (1.26–2.33)	1.52 (1.11–2.09)
No prevalent 9vHPV infection	9vHPV	1267	195	5.94	Ref	Ref	Ref
Prevalent 9vHPV infection	4vHPV	192	33	6.81	3.18 (.77–5.59)	1.88 (1.29–2.76)	1.75 (1.18–2.60)
No prevalent 9vHPV infection	4vHPV	1267	122	3.62	Ref	Ref	Ref
Prevalent 9vHPV infection	HPV16	192	11	2.15	0.31 (−1.03 to 1.66)	1.17 (.62–2.23)	1.16 (.60–2.24)
No prevalent 9vHPV infection	HPV16	1267	63	1.83	Ref	Ref	Ref
Prevalent 9vHPV infection	HPV16/18	192	19	3.77	1.31 (−.47 to 3.08)	1.53 (.93–2.52)	1.45 (.87–2.42)
No prevalent 9vHPV infection	HPV16/18	1267	84	2.46	Ref	Ref	Ref

Abbreviations: 9vHPV, 9-valent human papillomavirus; HM, heterosexual men; HPV, human papillomavirus; IRD, incidence rate difference; IRR, incidence rate ratio.

^a^Incidence rate ratio was adjusted for age, geographic region, and number of lifetime female sex partners.

In a separate analysis of MSM that included intra-anal sites of infection, the incidence (per 100 person-years) of incident-persistent 9vHPV infection was 20.45% among those with prevalent 9vHPV infection at baseline and 14.60% among those with no prevalent 9vHPV infection at baseline (Table 3). Incidence rates of incident-persistent 4vHPV infection were 9.67% among MSM with prevalent 9vHPV infection at baseline and 12.81% among those with no prevalent 9vHPV infection at baseline. Prevalent 9vHPV infection status at baseline was not associated with an increased risk of incident-persistent 9vHPV infection (IRD, 5.85 [95% CI, −1.50 to 13.20]; adjusted IRR, 0.99 [0.67–1.46]) or 4vHPV infection (IRD, −3.14 [95% CI, −8.62 to 2.34]), although a decreased risk of incident-persistent 4vHPV infection was observed (adjusted IRR, 0.53 [95% CI, .31–.89]).

**Table 3.** Incidence (per 100 Person-Years) of Incident-Persistent External Genital HPV Infection by Prevalent 9vHPV Infection Status at Baseline Among MSM (Aged 16–27 Years)

Prevalent 9vHPV Infection Status at Baseline	HPV Type Group of Incident-Persistent Infection	Participants at Risk, No.	Incident-Persistent Infections, No.	Incidence Per 100 Person-Years, %	IRD (95% CI)	IRR (95% CI)	Adjusted IRR^a^ (95% CI)
**MSM, excluding intra-anal infection (n = 260)**							
Prevalent 9vHPV infection	9vHPV	77	20	11.74	1.96 (−4.01 to 7.93)	1.20 (.72–2.01)	1.02 (.59–1.79)
No prevalent 9vHPV infection	9vHPV	183	40	9.78	Ref	Ref	Ref
Prevalent 9vHPV infection	4vHPV	77	10	5.57	−2.61 (−7.02 to 1.81)	0.68 (.34 to 1.37)	0.56 (.27–1.17)
No prevalent 9vHPV infection	4vHPV	183	34	8.18	Ref	Ref	Ref
Prevalent 9vHPV infection	HPV16	77	2	1.05	−1.90 (−4.07 to .26)	0.36 (.08–1.56)	0.22 (.05–1.02)
No prevalent 9vHPV infection	HPV16	183	13	2.95	Ref	Ref	Ref
Prevalent 9vHPV infection	HPV16/18	77	7	3.79	−0.63 (−4.06 to 2.81)	0.86 (.36–2.03)	0.64 (.25–1.61)
No prevalent 9vHPV infection	HPV16/18	183	19	4.42	Ref	Ref	Ref
**MSM, including intra-anal infection (n = 262)**							
Prevalent 9vHPV infection	9vHPV	101	42	20.45	5.85 (−1.50 to 13.20)	1.41 (.96–2.06)	0.99 (.67–1.46)
No prevalent 9vHPV infection	9vHPV	161	52	14.60	Ref	Ref	Ref
Prevalent 9vHPV infection	4vHPV	101	22	9.67	−3.14 (−8.62 to 2.34)	0.76 (.46–1.24)	0.53 (.31–.89)
No prevalent 9vHPV infection	4vHPV	161	46	12.81	Ref	Ref	Ref
Prevalent 9vHPV infection	HPV16	101	8	3.28	−1.06 (−4.14 to 2.01)	0.76 (.33–1.74)	0.51 (.21–1.24)
No prevalent 9vHPV infection	HPV16	161	17	4.35	Ref	Ref	Ref
Prevalent 9vHPV infection	HPV16/18	101	16	6.86	0.57 (−3.63 to 4.77)	1.10 (.59–2.05)	0.73 (.38–1.41)
No prevalent 9vHPV infection	HPV16/18	161	24	6.29	Ref	Ref	Ref

Abbreviations: 9vHPV, 9-valent human papillomavirus; HPV, human papillomavirus; IRD, incidence rate difference; IRR, incidence rate ratio; MSM, men who have sex with men.

^a^Incidence rate ratio was adjusted for age, geographic region, and number of lifetime male sex partners.

### Cumulative Incidence of Incident-Persistent 9-Valent Human Papillomavirus and 4-Valent Human Papillomavirus Infection by Prevalent Infection Status at Baseline

**Figure 1.**
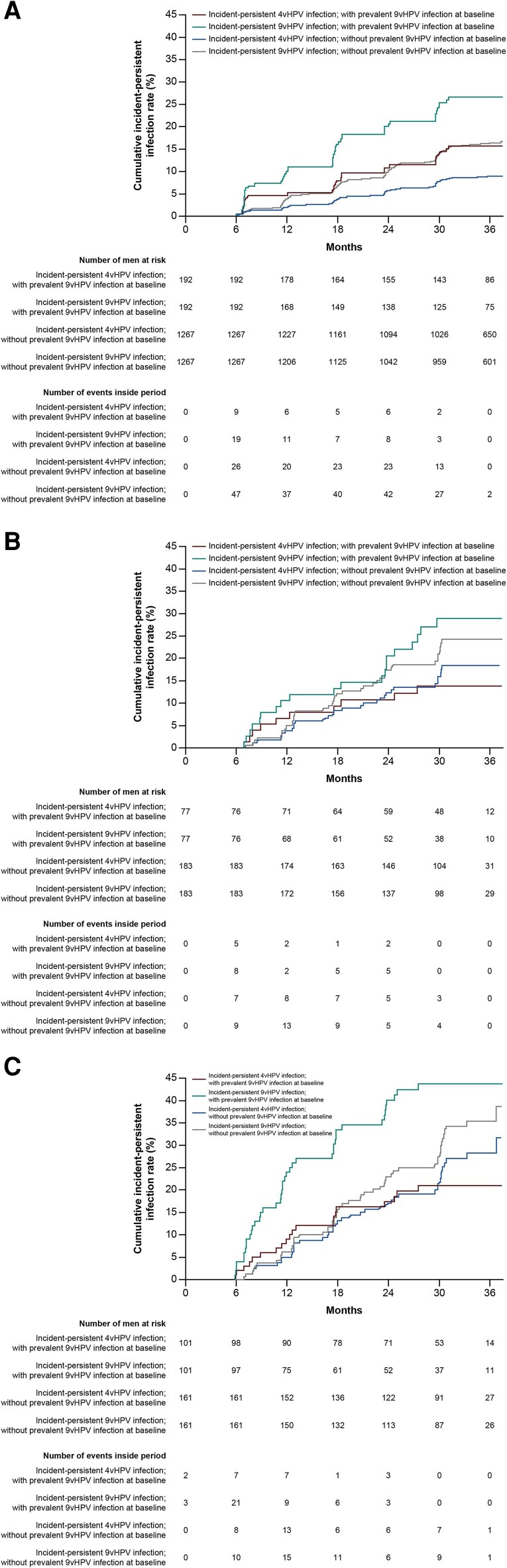
Kaplan–Meier estimates for the cumulative incidence of incident-persistent HPV infection by prevalent 9vHPV infection status at baseline among (*A*) HM, (*B*) MSM excluding intra-anal infections, and (*C*) MSM including intra-anal infections. Line color indicates the HPV type of incident-persistent HPV infection. 4vHPV, 4-valent human papillomavirus; 9vHPV, 9-valent human papillomavirus; HM, heterosexual men; HPV, human papillomavirus; MSM, men who have sex with men.

Among HM, cumulative incidence rates of incident-persistent 9vHPV and 4vHPV infections over 36 months were higher among those with prevalent 9vHPV infection at baseline than among those with no prevalent 9vHPV infection at baseline (9vHPV, 26.60% [95% CI, 20.74–33.74] vs 16.31% [95% CI, 14.31–8.55]; 4vHPV, 15.61% [95% CI, 11.04–21.83] vs 8.88% [95% CI, 7.39–10.66]) (Figure 1*A*; Supplementary Table 1). Cumulative incidence rates of incident-persistent HPV infection with HPV6, HPV11, HPV16, and HPV16/18 were not significantly different in HM compared with those with no prevalent 9vHPV infection at baseline (Supplementary Figure 1A; Supplementary Table 1).

Among MSM, cumulative incidence rates of incident-persistent 9vHPV and 4vHPV infections (excluding intra-anal infections) over 36 months were similar for those with and without prevalent 9vHPV infection at baseline (9vHPV, 28.89% [95% CI, 19.58–41.35] vs 24.22% [95% CI, 18.27–31.70]; 4vHPV, 13.83% [95% CI, 7.66–24.25] vs 18.37% [95% CI, 13.12–25.38]) (Figure 1*B*; Supplementary Table 2). Similarly, no statistically significant differences in the cumulative incidence of incident-persistent HPV infection were observed between those with and those without prevalent 9vHPV infection at baseline for HPV6, HPV11, HPV16, and HPV16/18 infections (Supplementary Figure 1B; Supplementary Table 2).

In a separate analysis of MSM that included the intra-anal infection site, cumulative incidence rates of incident-persistent 9vHPV and 4vHPV infections over 36 months were again similar for MSM with and without prevalent 9vHPV infection at baseline (9vHPV, 43.64% [95% CI, 34.33–54.25] vs 35.36% [95% CI, 27.96–44.04]; 4vHPV, 20.98% [95% CI, 14.04–30.68] vs 28.22% [95% CI, 21.35–36.73]) (Figure 1*C*; Supplementary Table 3). No statistically significant differences in cumulative incidence rates of incident-persistent HPV infection were observed between those with and those without prevalent 9vHPV infection at baseline for HPV6, HPV11, HPV16, and HPV16/18 infections (Supplementary Figure 1C; Supplementary Table 3).

## DISCUSSION

We focused this analysis on the association between prevalent 9vHPV infection status at baseline and the risk of incident-persistent 9vHPV infections among young HM and MSM. An association was observed for both 9vHPV and 4vHPV among HM but not among MSM. These results highlight that young HM with an existing HPV infection are at increased risk of additional infections compared with those without a prevalent infection. High cumulative incidence rates of incident-persistent 9vHPV infection were reported among MSM regardless of prevalent 9vHPV infection status at baseline, indicating that MSM, in general, experienced a high incidence of 9vHPV infection.

Our finding that prevalent 9vHPV infection was associated with future incident-persistent 9vHPV infection among HM adds to previous findings that are specific to same-type HPV reinfection in men. Analysis of data from 4123 unvaccinated men enrolled in the HIM study showed that detection of genital HPV infection was associated with an increased risk of reinfection with the same HPV type for at least 2 years [7]. In particular, an initial HPV16 infection was associated with a 20-fold increase in the risk of reinfection within 1 year [7]. In addition to new exposure from a sexual partner, autoinoculation or reactivation of a latent virus may contribute to detection of HPV infection in men who had a previous infection. For instance, increased risk of reinfection with the same HPV type was observed in both sexually active and celibate men in the HIM study [7]. In another analysis of men who have sex with women in the HIM study, previous genital HPV infection was associated with increased risk of a subsequent same-type anal 9vHPV infection that was unexplained by sexual intercourse with female partners, suggesting autoinoculation [12]. In additional studies it was reported that incident anal HPV detection was common in sexually inactive/nonexposed gay or bisexual MSM, supporting the idea that reactivation of a latent HPV infection could contribute to reinfection [13, 14]. Taken together, these findings suggest that both autoinoculation and reactivation of latent virus might play important roles in reinfection or redetection of infection, contributing to the incident-persistent 9vHPV infection quantified in the current analysis, as well as the overall burden of HPV infection among men.

MSM experienced high cumulative incidence rates of incident-persistent 9vHPV infection irrespective of prevalent 9vHPV infection status at baseline. This could be explained by the greater proportions of MSM, either with or without prevalent 9vHPV infection at baseline, who had 4 or 5 lifetime sexual partners (55.8% and 39.3%, respectively) compared to HM (47.4% and 28.8%, respectively). Greater exposure to HPV may therefore have occurred among MSM, increasing the risk of incident-persistent anogenital infections [15]. Additionally, in the current analysis, many MSM with or without baseline 9vHPV infection had 3–6 sex partners with whom they had insertive (55.8% and 36.6%, respectively) or receptive (48.1% and 37.2%, respectively) anal intercourse; previous research reported receptive anal intercourse as a risk factor for acquiring HPV [15, 16]. Similarly, previous analyses of the full HM and MSM cohorts in the V501-020 trial similarly identified a higher number of lifetime sexual partners as a risk factor for the detection of prevalent HPV infection in both HM and MSM [15, 17].

Because HPV vaccines provide maximum protection when administered before exposure and acquisition of HPV infection, in many countries, the recommended target population for routine HPV vaccination is children and adolescents (aged 9–14 years) [5, 6]. However, our findings highlight the benefit of vaccinating males who have not been vaccinated as young adolescents, including young adult HM who have an existing infection, as well as MSM more broadly, to prevent acquisition of additional new infections. Given that determining HPV infection status in clinical practice may be challenging, our study results support HPV vaccination for all young men aged 16 years or older who have not been vaccinated as young adolescents. Indeed, the US Advisory Committee on Immunization Practices recommends catch-up vaccination through age 26 years and vaccination of some people up to age 45 years [5]. Additionally, in an open-label, long-term extension of the V501-020 trial, catch-up vaccination reduced the incidence of anal lesions among young men exposed to HPV, further supporting the extension of HPV vaccination to all young adult men [18].

A methodological strength of the V501-020 trial is that HPV testing was conducted frequently (every 6 months). In addition, V501-020 comprised a large overall sample (N = 4065) of men enrolled from several countries across many geographical regions. However, the V501-020 trial restricted participants to no more than 5 lifetime sex partners, which may have selected for males with a lower likelihood of HPV exposure and thus may not be representative of the general population of sexually active men. Furthermore, although we observed a significant protective effect against incident-persistent 4vHPV infection among MSM with a prevalent 9vHPV infection at baseline when including intra-anal site infections (adjusted IRR, 0.53 [95% CI, .31–.89]), this may have occurred by chance, as the sample size was small, and we did not observe a similar trend for incident-persistent 9vHPV infection. Additionally, in our Kaplan–Meier analysis among MSM, including intra-anal site infections, we observed a high cumulative incidence of incident-persistent 9vHPV and 4vHPV infections over 36 months among MSM both with (9vHPV, 43.64%; 4vHPV, 20.98%) and without (9vHPV, 35.36%; 4vHPV, 28.22%) prevalent 9vHPV infection at baseline. Finally, the role that autoinoculation plays in reinfection could not be assessed in the current analysis.

## CONCLUSIONS

The findings of this study in a large global sample of unvaccinated young men directly show that HM who have an existing 9vHPV infection are at high risk of additional infection. Furthermore, MSM had high rates of anogenital 9vHPV infection irrespective of prevalent 9vHPV infection status at baseline, indicating that this is a high-risk population in general. Therefore, young men with a known HPV infection and those who do not have an infection (particularly in groups with higher likelihood of infection such as MSM) will benefit from vaccination. This highlights the importance of considering HPV vaccination for all young men up to age 45 years, regardless of previous HPV infection or associated disease.

## Supplementary Material

ofag045_Supplementary_Data

## Supplementary Data

Supplementary materials are available at *Online Forum Infectious Diseases* online. Consisting of data provided by the authors to benefit the reader, the posted materials are not copyedited and are the sole responsibility of the authors, so questions or comments should be addressed to the corresponding author.
