# Supplementary material for: High Risk of Incident-Persistent Human Papillomavirus Infection With 9-Valent Vaccine Types in Young Men With an HPV Infection
Source: Open Forum Infect Dis. 2026 Feb 2;13(3):ofag045. doi: 10.1093/ofid/ofag045 (PMC12955700; doi:10.1093/ofid/ofag045)

**Supplementary Index**

**Supplementary Table 1.** Kaplan-Meier Estimates for the Cumulative Incidence of Incident-Persistent^a^ HPV6, HPV11, or HPV16/18 Infection by Prevalent^b^ 9vHPV Infection Status at Baseline Among HM^c^

| **HPV Type** | **0 Months** | **6 Months** | **12 Months** | **18 Months** | **24 Months** | **30 Months** | **36 Months** |
| --- | --- | --- | --- | --- | --- | --- | --- |
| **With prevalent 9vHPV infection at baseline, % (95% confidence interval)** | | | | | | | |
| **HPV6** | 0.00 (0.00–0.00) | 0.00 (0.00–0.00) | 1.04 (0.26–4.10) | 3.82 (1.84–7.86) | 4.96 (2.61–9.33) | 6.15 (3.45–10.83) | 6.15 (3.45–10.83) |
| **HPV11** | 0.00 (0.00–0.00) | 0.00 (0.00–0.00) | 0.00 (0.00–0.00) | 0.56 (0.08–3.92) | 1.70 (0.55–5.19) | 1.70 (0.55–5.19) | 1.70 (0.55–5.19) |
| **HPV16** | 0.00 (0.00–0.00) | 0.52 (0.07–3.64) | 2.60 (1.09–6.14) | 2.60 (1.09–6.14) | 3.17 (1.44–6.92) | 4.37 (2.21–8.57) | 4.98 (2.62–9.38) |
| **HPV16/18** | 0.00 (0.00–0.00) | 0.52 (0.07–3.64) | 3.65 (1.75–7.49) | 3.65 (1.75–7.49) | 4.21 (2.13–8.24) | 6.60 (3.80–11.36) | 7.82 (4.70–12.87) |
| **4vHPV types** | 0.00 (0.00–0.00) | 0.52 (0.07–3.64) | 4.69 (2.47–8.81) | 8.00 (4.90–12.93) | 10.83 (7.12–16.29) | 14.40 (10.03–20.45) | 15.61 (11.04–21.83) |
| **9vHPV types** | 0.00 (0.00–0.00) | 0.52 (0.07–3.64) | 9.94 (6.46–15.14) | 16.03 (11.49–22.13) | 20.01 (14.91–26.56) | 24.78 (19.11–31.77) | 26.60 (20.74–33.74) |
| **With no prevalent 9vHPV infection at baseline, % (95% confidence interval)** | | | | | | | |
| **HPV6** | 0.00 (0.00–0.00) | 0.08 (0.01–0.56) | 0.55 (0.26–1.16) | 0.88 (0.49–1.58) | 1.90 (1.27–2.85) | 2.53 (1.77–3.60) | 2.81 (2.00–3.93) |
| **HPV11** | 0.00 (0.00–0.00) | 0.00 (0.00–0.00) | 0.00 (0.00–0.00) | 0.00 (0.00–0.00) | 0.09 (0.01–0.61) | 0.44 (0.18–1.05) | 0.53 (0.24–1.17) |
| **HPV16** | 0.00 (0.00–0.00) | 0.00 (0.00–0.00) | 1.11 (0.66–1.86) | 2.17 (1.49–3.15) | 2.59 (1.84–3.65) | 3.66 (2.73–4.88) | 4.30 (3.28–5.63) |
| **HPV16/18** | 0.00 (0.00–0.00) | 0.00 (0.00–0.00) | 1.50 (0.96–2.35) | 2.81 (2.02–3.89) | 3.65 (2.74–4.86) | 4.72 (3.66–6.07) | 5.55 (4.38–7.01) |
| **4vHPV types** | 0.00 (0.00–0.00) | 0.08 (0.01–0.56) | 2.06 (1.40–3.00) | 3.69 (2.77–4.89) | 5.64 (4.48–7.09) | 7.68 (6.30–9.34) | 8.88 (7.39–10.66) |
| **9vHPV types** | 0.00 (0.00–0.00) | 0.08 (0.01–0.56) | 3.72 (2.81–4.92) | 6.81 (5.55–8.36) | 10.12 (8.56–11.95) | 13.83 (11.99–15.92) | 16.31 (14.31–18.55) |

The analysis population for Kaplan-Meier estimates comprised participants who were randomly assigned to the placebo arm of the V501-020 trial, had a valid day 1 PCR result for all 9vHPV types from swabbed specimens at each anogenital site, and had at least 2 follow-up visits with a PCR test result for any 9vHPV type (6/11/16/18/31/33/45/52/58).

Abbreviations: 9vHPV, 9-valent HPV vaccine; HM, heterosexual male; HPV, human papillomavirus.

^a^Incident-persistent HPV infection was defined as a new HPV type at follow-up that was not present at baseline at the same anatomical site.

^b^Prevalent 9vHPV infection was defined as any 9vHPV at baseline.

^c^Anatomical sites among HM included penile/scrotal and perineal/perianal sites.

**Supplementary Table 2.** Kaplan-Meier Estimates for the Cumulative Incidence of Incident-Persistent^a^ HPV6, HPV11, or HPV16/18 Infection by Prevalent^b^ 9vHPV Infection Status at Baseline Among MSM Excluding Intra-Anal Infections^d^

| **HPV Type** | **0 Months** | **6 Months** | **12 Months** | **18 Months** | **24 Months** | **30 Months** | **36 Months** |
| --- | --- | --- | --- | --- | --- | --- | --- |
| **With prevalent 9vHPV infection at baseline, % (95% confidence interval)** | | | | | | | |
| **HPV6** | 0.00 (0.00–0.00) | 0.00 (0.00–0.00) | 2.63 (0.66–10.11) | 3.95 (1.29–11.74) | 5.36 (2.04–13.66) | 5.36 (2.04–13.66) | 5.36 (2.04–13.66) |
| **HPV11** | 0.00 (0.00–0.00) | 0.00 (0.00–0.00) | 1.32 (0.19–8.97) | 1.32 (0.19–8.97) | 1.32 (0.19–8.97) | 1.32 (0.19–8.97) | 1.32 (0.19–8.97) |
| **HPV16** | 0.00 (0.00–0.00) | 0.00 (0.00–0.00) | 0.00 (0.00–0.00) | 0.00 (0.00–0.00) | 0.00 (0.00–0.00) | 1.49 (0.21–10.13) | 1.49 (0.21–10.13) |
| **HPV16/18** | 0.00 (0.00–0.00) | 0.00 (0.00–0.00) | 2.63 (0.66–10.11) | 4.00 (1.31–11.90) | 4.00 (1.31–11.90) | 7.13 (3.02–16.36) | 7.13 (3.02–16.36) |
| **4vHPV types** | 0.00 (0.00–0.00) | 0.00 (0.00–0.00) | 6.58 (2.79–15.09) | 9.27 (4.53–18.47) | 10.69 (5.49–20.25) | 13.83 (7.66–24.25) | 13.83 (7.66–24.25) |
| **9vHPV types** | 0.00 (0.00–0.00) | 0.00 (0.00–0.00) | 10.53 (5.41–19.95) | 13.22 (7.34–23.18) | 20.51 (12.88–31.74) | 28.89 (19.58–41.35) | 28.89 (19.58–41.35) |
| **With no prevalent 9vHPV infection at baseline, % (95% confidence interval)** | | | | | | | |
| **HPV6** | 0.00 (0.00–0.00) | 0.00 (0.00–0.00) | 1.10 (0.28–4.31) | 3.32 (1.50–7.23) | 5.05 (2.66–9.49) | 7.84 (4.60–13.20) | 8.66 (5.18–14.29) |
| **HPV11** | 0.00 (0.00–0.00) | 0.00 (0.00–0.00) | 0.55 (0.08–3.82) | 1.10 (0.28–4.32) | 2.83 (1.19–6.67) | 2.83 (1.19–6.67) | 2.83 (1.19–6.67) |
| **HPV16** | 0.00 (0.00–0.00) | 0.00 (0.00–0.00) | 0.55 (0.08–3.82) | 1.66 (0.54–5.07) | 2.25 (0.85–5.88) | 3.05 (1.27–7.26) | 4.68 (2.23–9.69) |
| **HPV16/18** | 0.00 (0.00–0.00) | 0.00 (0.00–0.00) | 2.19 (0.83–5.72) | 3.86 (1.86–7.92) | 4.44 (2.25–8.69) | 5.24 (2.74–9.89) | 6.86 (3.81–12.17) |
| **4vHPV types** | 0.00 (0.00–0.00) | 0.00 (0.00–0.00) | 3.83 (1.84–7.86) | 8.26 (5.06–13.33) | 12.31 (8.28–18.10) | 15.91 (11.14–22.44) | 18.37 (13.12–25.38) |
| **9vHPV types** | 0.00 (0.00–0.00) | 0.00 (0.00–0.00) | 4.92 (2.59–9.25) | 12.14 (8.16–17.85) | 17.35 (12.53–23.76) | 20.95 (15.53–27.94) | 24.22 (18.27–31.70) |

The analysis population for Kaplan-Meier estimates comprised participants who were randomly assigned to the placebo arm of the V501-020 trial, had a valid day 1 PCR result for all 9vHPV types from swabbed specimens at each anogenital site, and had at least 2 follow-up visits with a PCR test result for any 9vHPV type (6/11/16/18/31/33/45/52/58).

Abbreviations: 9vHPV, 9-valent HPV vaccine; HPV, human papillomavirus; MSM, men who have sex with men.

^a^Incident-persistent HPV infection was defined as a new HPV type at follow-up that was not present at baseline at the same anatomical site.

^b^Prevalent 9vHPV infection was defined as any 9vHPV at baseline.

^c^Anatomical sites among MSM included penile/scrotal and perineal/perianal sites (intra-anal sites were excluded).

**Supplementary Table 3.** Kaplan-Meier Estimates for the Cumulative Incidence of Incident-Persistent^a^ HPV6, HPV11, or HPV16/18 Infection by Prevalent^b^ 9vHPV Infection Status at Baseline Among MSM Including Intra-Anal Infections^e^

| **HPV Type** | **0 Months** | **6 Months** | **12 Months** | **18 Months** | **24 Months** | **30 Months** | **36 Months** |
| --- | --- | --- | --- | --- | --- | --- | --- |
| **With prevalent 9vHPV infection at baseline, % (95% confidence interval)** | | | | | | | |
| **HPV6** | 0.00 (0.00–0.00) | 1.00 (0.14–6.89) | 3.00 (0.98–9.01) | 4.01 (1.52–10.33) | 5.11 (2.16–11.86) | 5.11 (2.16–11.86) | 5.11 (2.16–11.86) |
| **HPV11** | 0.00 (0.00–0.00) | 0.00 (0.00–0.00) | 1.00 (0.14–6.89) | 1.00 (0.14–6.89) | 1.00 (0.14–6.89) | 2.14 (0.54–8.31) | 2.14 (0.54–8.31) |
| **HPV16** | 0.00 (0.00–0.00) | 1.00 (0.14–6.89) | 1.00 (0.14–6.89) | 3.06 (1.00–9.20) | 3.06 (1.00–9.20) | 5.50 (2.31–12.77) | 5.50 (2.31–12.77) |
| **HPV16/18** | 0.00 (0.00–0.00) | 1.00 (0.14–6.89) | 5.00 (2.11–11.60) | 11.21 (6.37–19.34) | 11.21 (6.37–19.34) | 13.65 (8.14–22.41) | 13.65 (8.14–22.41) |
| **4vHPV types** | 0.00 (0.00–0.00) | 2.00 (0.50–7.76) | 9.00 (4.79–16.58) | 16.24 (10.27–25.14) | 17.35 (11.16–26.44) | 20.98 (14.04–30.68) | 20.98 (14.04–30.68) |
| **9vHPV types** | 0.00 (0.00–0.00) | 4.00 (1.52–10.31) | 24.00 (16.78–33.63) | 33.40 (25.03–43.65) | 40.03 (31.03–50.52) | 43.64 (34.33–54.25) | 43.64 (34.33–54.25) |
| **With no prevalent 9vHPV infection at baseline, % (95% confidence interval)** | | | | | | | |
| **HPV6** | 0.00 (0.00–0.00) | 0.00 (0.00–0.00) | 2.48 (0.94–6.48) | 6.26 (3.42–11.32) | 6.91 (3.88–12.13) | 9.98 (6.11–16.07) | 11.72 (7.41–18.30) |
| **HPV11** | 0.00 (0.00–0.00) | 0.00 (0.00–0.00) | 0.62 (0.09–4.33) | 1.88 (0.61–5.70) | 3.20 (1.34–7.53) | 3.20 (1.34–7.53) | 3.20 (1.34–7.53) |
| **HPV16** | 0.00 (0.00–0.00) | 0.00 (0.00–0.00) | 0.62 (0.09–4.33) | 1.88 (0.61–5.71) | 3.84 (1.74–8.36) | 5.41 (2.73–10.59) | 9.33 (5.31–16.12) |
| **HPV16/18** | 0.00 (0.00–0.00) | 0.00 (0.00–0.00) | 1.86 (0.60–5.67) | 5.01 (2.54–9.77) | 6.98 (3.92–12.25) | 8.54 (5.03–14.31) | 13.37 (8.51–20.67) |
| **4vHPV types** | 0.00 (0.00–0.00) | 0.00 (0.00–0.00) | 4.97 (2.52–9.69) | 13.13 (8.77–19.43) | 17.06 (12.03–23.90) | 21.70 (15.89–29.22) | 28.22 (21.35–36.73) |
| **9vHPV types** | 0.00 (0.00–0.00) | 0.00 (0.00–0.00) | 6.21 (3.39–11.24) | 15.64 (10.85–22.26) | 22.85 (17.05–30.25) | 27.42 (21.01–35.31) | 35.36 (27.96–44.04) |

The analysis population for Kaplan-Meier estimates comprised participants who were randomly assigned to the placebo arm of the V501-020 trial, had a valid day 1 PCR result for all 9vHPV types from swabbed specimens at each anogenital site, and had at least 2 follow-up visits with a PCR test result for any 9vHPV type (6/11/16/18/31/33/45/52/58).

Abbreviations: 9vHPV, 9-valent HPV vaccine; HPV, human papillomavirus; MSM, men who have sex with men.

^a^Incident-persistent HPV infection was defined as a new HPV type at follow-up that was not present at baseline at the same anatomical site.

^b^Prevalent 9vHPV infection was defined as any 9vHPV at baseline.

^c^Anatomical sites among MSM in a separate analysis included penile/scrotal, perineal/perianal, and intra-anal sites.

**Supplementary Figure 1.** Kaplan-Meier estimates for the cumulative incidence of incident-persistent^a^ HPV6, HPV11, or HPV16/18 infection by prevalent^b^ 9vHPV infection status at baseline among (*A*) HM,^c^ (*B*) MSM excluding intra-anal infections,^d^ and (*C*) MSM including intra-anal infections.^e^ Line color indicates the HPV type of incident-persistent HPV infection. 9vHPV, 9-valent HPV vaccine; HM, heterosexual male; HPV, human papillomavirus; MSM, men who have sex with men.

The analysis population for Kaplan-Meier estimates comprised participants who were randomly assigned to the placebo arm of the V501-020 trial, had a valid day 1 PCR result for all 9vHPV types from swabs at each anogenital site, and had at least 2 follow-up visits with a PCR test result for any 9vHPV type (6/11/16/18/31/33/45/52/58).

^a^Incident-persistent HPV infection was defined as a new HPV type at follow-up not present at baseline at the same anatomic site.

^b^Prevalent 9vHPV infection was defined as any 9vHPV at baseline.

^c^Anatomical sites among HM included penile/scrotal and perineal/perianal sites.

^d^Anatomical sites among MSM included penile/scrotal and perineal/perianal sites (intra-anal sites were excluded).

^e^Anatomical sites among MSM in a separate analysis included penile/scrotal, perineal/perianal, and intra-anal sites.


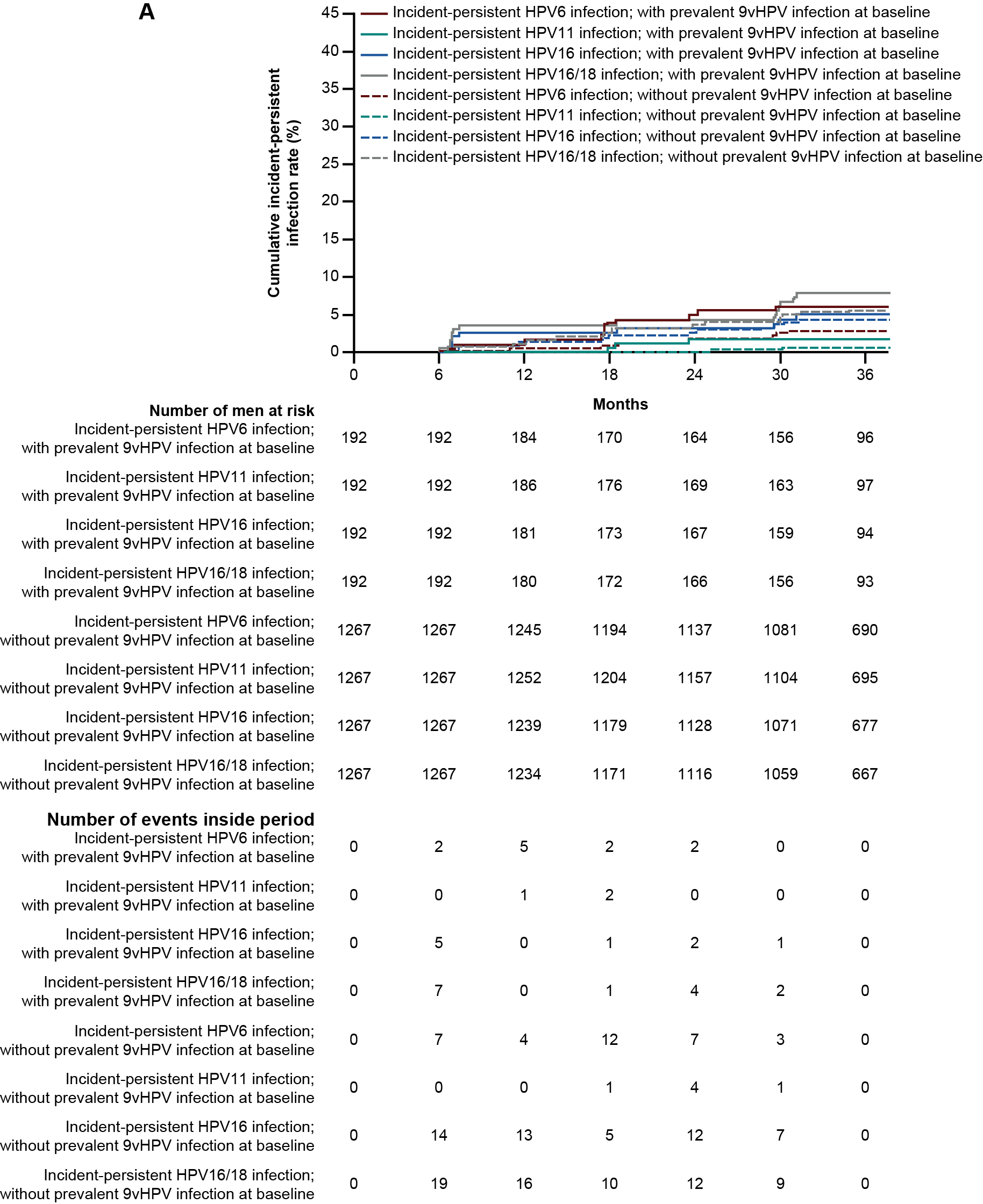


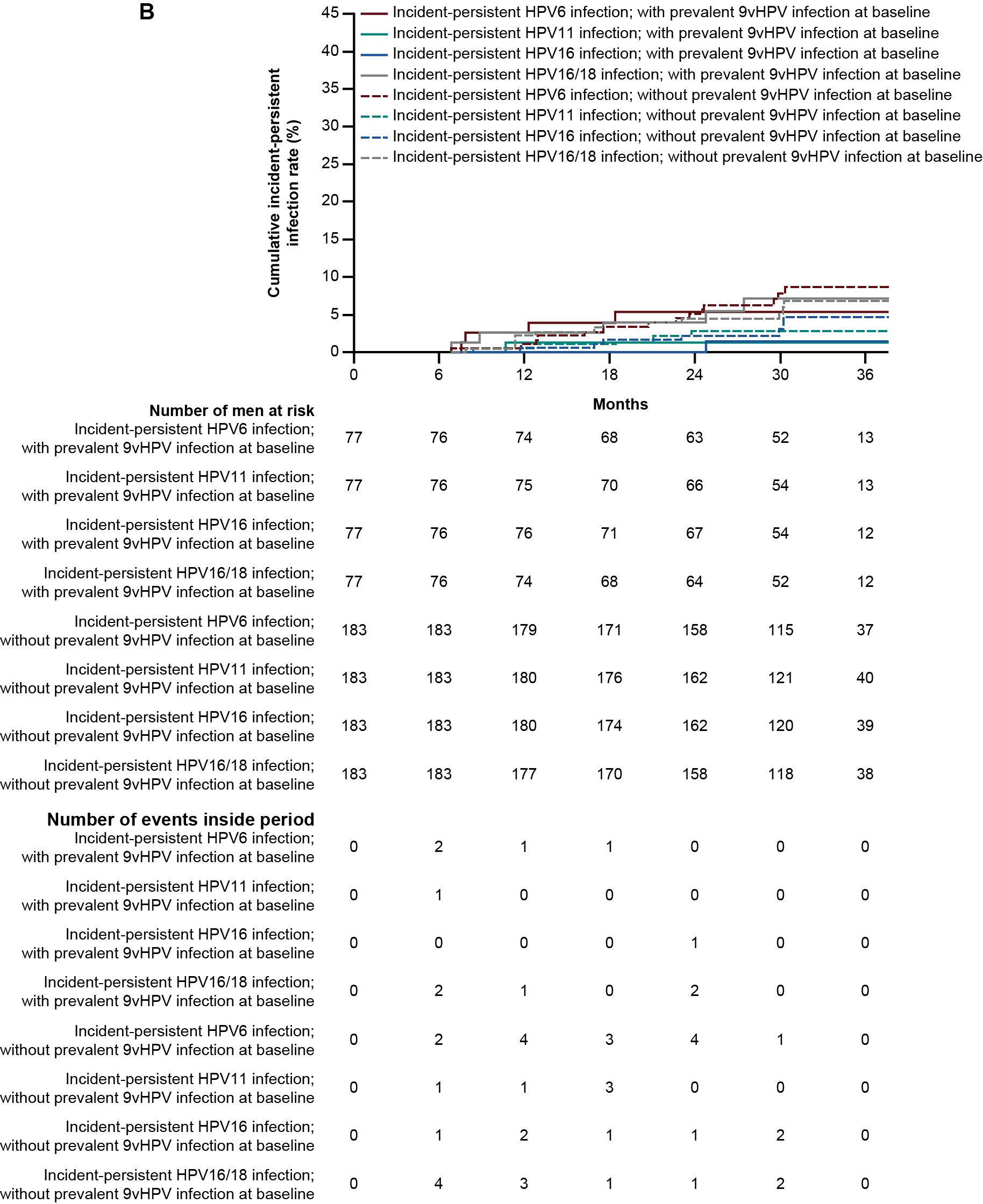


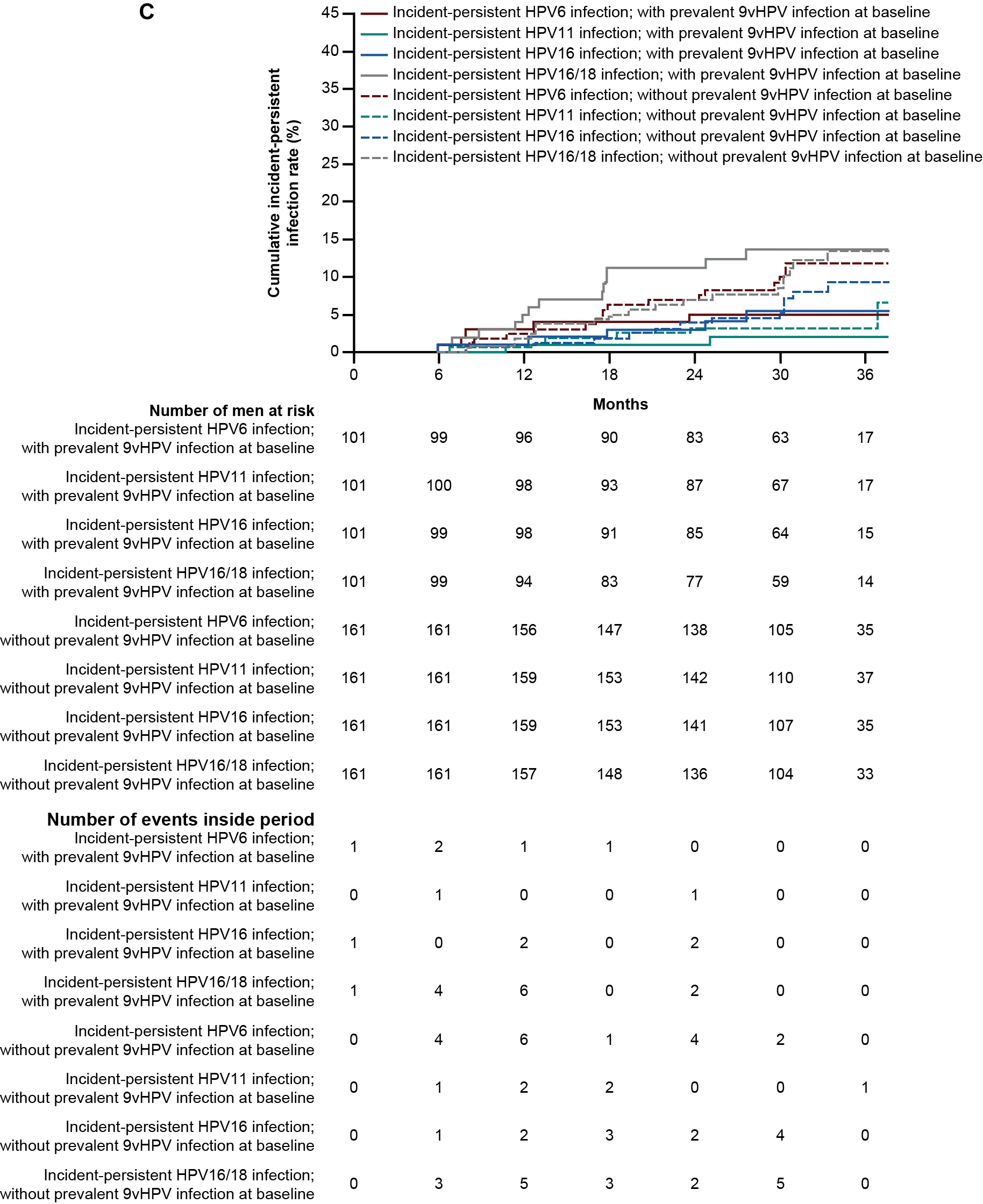

Supplement: ofag045_Supplementary_Data [file ofag045_supplementary_data.docx]
